# Delta Shock Index During Emergency Department Stay Is Associated With in Hospital Mortality in Critically Ill Patients

**DOI:** 10.3389/fmed.2021.648375

**Published:** 2021-04-22

**Authors:** Yi-Syun Huang, I-Min Chiu, Ming-Ta Tsai, Chun-Fu Lin, Chien-Fu Lin

**Affiliations:** ^1^Department of Emergency Medicine, Kaohsiung Chang Gung Memorial Hospital, Kaohsiung, Taiwan; ^2^Department of Computer Science and Engineering, National Sun Yet-sen University, Kaohsiung, Taiwan

**Keywords:** delta shock index, emergency department, mortality, critical ill, intensive care unit

## Abstract

**Background:** Delta shock index (SI; i.e., change in SI over time) has been shown to predict mortality and need for surgical intervention among trauma patients at the emergency department (ED). However, the usefulness of delta SI for prognosis assessment in non-traumatic critically ill patients at the ED remains unknown. The aim of this study was to analyze the association between delta SI during ED management and in-hospital outcomes in patients admitted to the intensive care unit (ICU).

**Method:** This was a retrospective study conducted in two tertiary medical centers in Taiwan from January 1, 2016, to December 31, 2017. All adult non-traumatic patients who visited the ED and who were subsequently admitted to the ICU were included. We calculated delta SI by subtracting SI at ICU admission from SI at ED triage, and we analyzed its association with in-hospital outcomes. SI was defined as the ratio of heart rate to systolic blood pressure (SBP). The primary outcome was in-hospital mortality, and the secondary outcomes were hospital length of stay (HLOS) and early mortality. Early mortality was defined as mortality within 48 h of ICU admission.

**Result:** During the study period, 11,268 patients met the criteria and were included. Their mean age was 64.5 ± 15.9 years old. Overall, 5,830 (51.6%) patients had positive delta SI. Factors associated with a positive delta SI were multiple comorbidities (51.2% vs. 46.3%, *p* < 0.001) and high Simplified Acute Physiology Score [39 (29–51) vs. 37 (28–47), *p* < 0.001). Patients with positive delta SI were more likely to have tachycardia, hypotension, and higher SI at ICU admission. In the regression analysis, high delta SI was associated with in-hospital mortality [aOR (95% CI): 1.21 (1.03–1.42)] and early mortality [aOR (95% CI): 1.26 (1.07–1.48)], but not for HLOS [difference (95% CI): 0.34 (−0.48 to 1.17)]. In the subgroup analysis, high delta SI had higher odds ratios for both mortality and early mortality in elderly [aOR (95% CI): 1.59 (1.11–2.29)] and septic patients [aOR (95% CI): 1.54 (1.13–2.11)]. It also showed a higher odds ratio for early mortality in patients with triage SBP <100 mmHg [aOR (95% CI): 2.14 (1.21–3.77)] and patients with triage SI ≥ 0.9 [aOR (95% CI): 1.62 (1.01–2.60)].

**Conclusion:** High delta SI during ED stay is correlated with in-hospital mortality and early mortality in patients admitted to the ICU via ED. Prompt resuscitation should be performed, especially for those with old age, sepsis, triage SBP <100 mmHg, or triage SI ≥ 0.9.

## Introduction

In the emergency department (ED), the survival rate of patients is mainly determined by the severity of acute illness on admission (1, 2) and the quality of care throughout the treatment process (3). Numerous scoring systems based on physiological parameters recorded in the ED have been developed for initial patient assessment and the identification of patients at risk (4–7). Nevertheless, patient deterioration and unexpected death are often preceded by abnormalities in vital signs in the ED (8, 9). It is important to document vital sign changes in the ED for physicians to provide adequate management.

Shock index (SI), calculated from the two most commonly used physiological measures [heart rate (HR) divided by systolic blood pressure (SBP)], is a simple bedside assessment originally developed to evaluate the degree of shock in hemorrhagic and septic patients (10). In recent studies, it has been used for the prediction of outcomes in other critically ill patients, including those with severe sepsis (11, 12), hemorrhagic shock (13), pulmonary embolism (14), and acute myocardial infarction (15). An SI <0.9 is considered to be associated with increased mortality risk (16). This cutoff value of the SI may help with early mobilization of resources in the ED.

Recently, it has been noted that delta SI (i.e., change of SI over time) predicts mortality in apparently hemodynamically stable trauma patients with normal traditional vital signs in the ED (17, 18). A similar result has been observed for postpartum hemorrhage in the ED; delta SI was superior in identifying the need for emergent intervention than other traditional vital signs (19).

However, research on the prognosis value of delta SI in critically ill patients in the ED is scarce. The aim of this study was to analyze the association between delta SI in the ED and in-hospital outcomes of critically ill patients who required intensive care unit (ICU) admission. Having a simple index that reliably correlates pre-ICU admission physiological parameters to mortality would be ideal to assess the quality of care in critically ill patients.

## Method

This was a retrospective database study conducted in two tertiary medical centers in Taiwan from January 1, 2016, to December 31, 2017. One hospital was located in northern Taiwan and the other in southern Taiwan, and they were both the largest medical centers in their metropolitan areas. The study protocol was approved by the institutional review board of both hospitals (IRB number 202002043B0; date of approval, December 1, 2020). All patients' and physicians' records and information were anonymized and de-identified before analysis.

**Figure 1:**
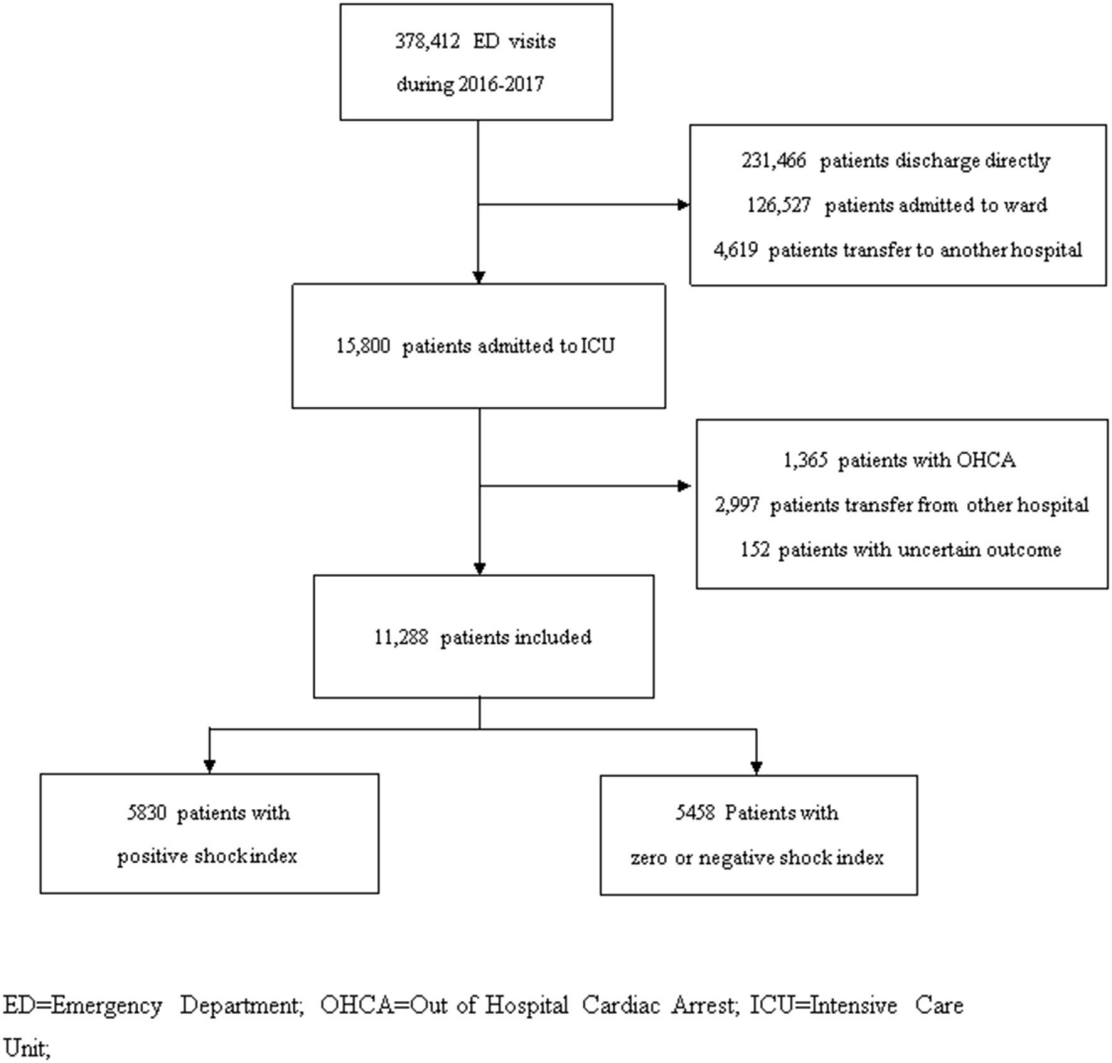
Patient inclusion flowchart in studied hospital during 2016–2017. ED, Emergency Department; OHCA, Out of Hospital Cardiac Arrest; ICU, Intensive Care Unit.

All adult non-traumatic patients visiting the ED and subsequently admitted to the ICU were included. Patients with uncertain outcomes (discharged against medical advice and transferred to another hospital), transferred from other hospitals, presented with out-of-hospital cardiac arrest, or deceased at the ED were excluded (Figure 1). Patients' demographic data (age and sex), underlying comorbidities, vital signs at triage and at ICU admission, laboratory tests, and diagnosis at ICU admission were extracted from the electronic medical records of the studied hospitals for analysis. The Simplified Acute Physiology Score (SAPS) was computed based on the collected parameters for severity evaluation (20).

We calculated SI, defined as the ratio of HR to SBP, from vital signs at ED triage and at ICU admission. Delta SI was calculated by subtracting SI at ICU admission from SI at ED triage. Patients were then divided into two groups (patients with a positive delta SI and patients with zero or negative delta SI), and their demographics and clinical characteristics were compared. The primary outcome was in-hospital mortality, and the secondary outcomes were hospital length of stay (HLOS) and early mortality. Early mortality was defined as mortality within 48 h of ICU admission. We also performed subgroup analysis based on patient's age, comorbidity, vital signs, and diagnostic categories to clarify the association of delta SI to patient's outcome in different clinical conditions. Age older than 65 years was considered as elderly.

Data are presented as the mean (standard deviation) for continuous variables, proportions for nominal variables, and median (interquartile range) for ordinal variables. We performed Student's *t*-test and chi square analysis to determine the parameters that correlated with positive delta SI and with zero or negative SI. Logistic regressions assessing the association of clinical outcomes with delta SI were performed after adjusting for confounding factors. A two-sided *p* < 0.05 was considered statistically significant. Stratified regression analyses assessing the relationship between delta SI and clinical outcomes in different ages, comorbidities, vital signs, and diagnosis categories were also performed. All statistical analyses were conducted using IBM SPSS Statistics for Mac (Version 26).

## Result

During the study period, 11,268 patients who met the criteria were included. Their mean age was 64.5 ± 15.9 years, and 64.3% were male. The average SAPS was 38 (29–49). Of all patients, 5,830 (51.6%) had a positive delta SI. The parameters significantly associated with positive delta SI were multiple comorbidities (51.2% vs. 46.3%, *p* < 0.001) and high SAPS [39 (29–51) vs. 37 (28–47), *p* < 0.001]. In addition, compared with patients with zero or negative delta SI, patients with positive delta SI were less likely to have tachycardia, hypotension, and high SI at ED triage. Conversely, they were more likely to present with tachycardia, hypotension, and high SI at ICU admission (Table 1). Regarding prognosis at the ICU (Table 2), positive delta SI was significantly associated with higher mortality (20.3 vs. 18.9%, *p* = 0.032) and early mortality (6.5 vs. 5.2%, *p* = 0.005) than was zero or negative delta SI, while no significant relationship was observed with HLOS (13 vs. 13 days, *p* = 0.277).

**Table 1:** Demographic and clinical characteristics in comparison of positive delta SI with zero or negative delta SI.

**Variables**	**Positive delta SI (*n* = 5,830)**	**Zero or negative delta SI (*n* = 5,458)**	***p*-value**
Age, year, median (IQR)	65 (54–76)	66 (54–77)	0.984
Male sex, %	64.0	64.6	0.467
Comorbidity≥2, %	51.2	46.3	<0.001
ED LOS, hours, median (IQR)	12.1 (6.3-18.1)	11.9 (6.1-17.7)	0.639
at ED Triage, mean (SD)			
Heart rate	90 (24.1)	104 (25.8)	<0.001
SBP	158 (37.0)	124 (33.5)	<0.001
DBP	90 (22.5)	75 (22.8)	<0.001
SI	0.60 (0.35)	0.91 (0.31)	<0.001
at ICU admission, mean (SD)			
Heart rate	94 (21.5)	89 (21.5)	<0.001
SBP	128 (26.6)	134 (26.9)	<0.001
DBP	74 (17.6)	76 (17.1)	<0.001
SI	0.78 (0.24)	0.69 (0.25)	<0.001
Severity score, median (IQR)			
SAPS	39 (29-51)	37 (28-47)	<0.001
In-hospital outcome			
Mortality, %	20.3	18.9	0.032
Mortality in 48 h, %	6.5	5.2	0.005
HLOS, d, median (IQR)	13 (7-23)	13 (7-23)	0.277
Comorbidity, %			
Hypertension	42.1	33.7	<0.001
Diabetes mellitus	23.0	22.4	0.449
Heart failure	13.5	14.5	0.128
Liver cirrhosis	6.9	9.8	<0.001
End stage renal disease	8.2	8.0	0.629
Malignancy	11.4	14.6	<0.001
Old stroke	30.7	22.8	<0.001

*SI, Shock Index; IQR, Interquartile Range; SD, Standard Deviation; ED LOS, Emergency Department Length Of Stay; SBP, Systolic Blood Pressure; DBP, Diastolic Blood Pressure; SAPS, Simplified Acute Physiology Score; HLOS, Hospital Length Of Stay*.

**Table 2:** Logistic Regression analysis of delta Shock Index to in-hospital outcome.

	**aOR (95% CI)**	***p*-value**
Mortality	1.21 (1.03–1.42)	0.021
Early mortality	1.49 (1.13–1.96)	0.005
HLOS, d	0.34 (−0.48–1.17)	0.417

^*^logistic regression analysis performed by adjusting confounding factors include age, sex, comorbidities and SAPS.

*SI, Shock Index; HLOS, Hospital Length Of Stay; aOR, adjusted Odds Ratio*.

On binary logistic regression analysis, highly positive delta SI was an independent risk factor for in-hospital mortality [adjusted odds ratio (95% CI): 1.21 (1.03–1.42)] and early mortality [adjusted odds ratio (95% CI): 1.26 (1.07–1.48)]. On the other hand, the linear regression analysis on the association of delta SI with HLOS showed no significant difference [difference (95% CI): 0.34 (−0.48 to 1.17); Table 3].

**Table 3:** Subgroup regression analysis of delta SI to in-hospital mortality and early mortality.

	**Mortality**		**Early mortality**	
	**aOR (95% CI)**	***p*-value**	**aOR (95% CI)**	***p*-value**
**Age**				
≥65	1.59 (1.11–2.29)	0.012	1.66 (1.06–2.38)	0.013
18-64	1.02 (0.71–1.34)	0.875	1.65 (0.91–2.98)	0.098
Comorbidity≥2	1.09 (0.76–1.56)	0.657	1.45 (0.76–2.79)	0.260
**Vital sign**				
Triage SBP≥100	1.05 (0.74–1.49)	0.802	1.19 (0.65–2.17)	0.572
Triage SBP <100	1.03 (0.65–1.61)	0.908	2.14 (1.21–3.77)	0.009
Triage SI ≥ 0.9	0.95 (0.64–1.39)	0.773	1.62 (1.01–2.60)	0.038
Triage SI <0.9	1.07 (0.82–1.39)	0.641	1.13 (0.73–1.74)	0.594
**Diagnosis**				
Sepsis	1.54 (1.13–2.11)	0.007	1.46 (1.03–1.94)	0.033
Respiratory failure	0.97 (0.85–1.11)	0.645	1.06 (0.86–1.29)	0.596
Heart failure	1.22 (0.86–1.73)	0.260	1.64 (0.90–3.00)	0.108

^*^logistic regression analysis performed by adjusting confounding factors include age, sex, comorbidities and SAPS.

*SI, Shock Index; SBP, Systolic Blood Pressure; aOR, adjusted Odds Ratio*.

In the subgroup analysis, high delta SI had higher odds ratios for mortality in elderly patients [adjusted odds ratio (95% CI): 1.59 (1.11–2.29)] and in patients with a diagnosis of sepsis [adjusted odds ratio (95% CI): 1.54 (1.13–2.11)], with respect to other age ranges and diagnoses, respectively. The analysis also showed higher odds ratios for early mortality in elderly patients [adjusted odds ratio (95% CI): 1.66 (1.06–2.38)], in patients with triage SBP <100 [adjusted odds ratio (95% CI): 2.14 (1.21–3.77)], in patients with triage SI ≥ 0.9 [adjusted odds ratio (95% CI): 1.62 (1.01–2.60)], and in patients with a diagnosis of sepsis [adjusted odds ratio (95% CI): 1.46 (1.03–1.94)], compared with the other possibilities of each category. There were no statistical differences in the regression analyses regarding patients with a diagnosis of respiratory failure and heart failure in either of the two in-hospital outcomes with previous significant relationships.

## Discussion

The aim of this study was to determine the relationship between delta SI during ED management and in-hospital outcomes in patients admitted to the ICU via ED. In this study, we found that positive delta SI is more likely to occur in patients with multiple comorbidities and in patients who present at the ED with high SAPS, as shown in Table 1. There is no doubt that comorbidity is an important factor in estimating a patient's outcome; and in some cases, the patient's comorbid condition presents a greater risk than the index disease (21). Therefore, the association between delta SI and comorbidity was foreseeable: patients presenting to the ED with more comorbidities are at greater risk of deterioration. A similar conclusion can be drawn for patients with scores that indicate severe conditions, which were often admitted to the ICU. SAPS (20), which includes items such as age, physiological parameters, type of admission, and chronic diseases, is a reliable indicator of the risk of death upon ICU admission. Patients with high SAPS, which theoretically indicates poor outcome for patients admitted to the ICU, tended to have positive delta SI.

Moreover, the positive delta SI group had better initial vital signs than the zero or negative delta SI group. For this paradoxical phenomenon, ED clinicians usually spend more time and effort managing patients with worse vital signs. And this condition may lead to delayed assessment and treatment or to relatively conservative treatment in this group of patients with initially better vital signs at ED assessment (22, 23). In addition, patients in the negative delta SI group presented with higher SI (mean: 0.91) at ED triage, so they required aggressive and fast management due to their initially unstable conditions. Thus, more effort (continuous bedside evaluation, resuscitation, and re-evaluation) was devoted to them (24–26). Intubation, ventilation, volume support, and even vasoactive therapy were initiated earlier in the group of patients with worse vital signs, leading to negative delta SI in this group of patients.

Regarding the association between delta SI and in-hospital outcomes, there was a statistically significant difference between positive delta SI (worsened SI) and zero or negative delta SI (improved SI) in mortality and early mortality. Previous studies have demonstrated that positive delta SI during ED management is a strong predictor of mortality and of need for blood product transfusion in trauma patients (27). Regarding the connection between delta SI and HLOS, there was no statistically significant difference between the positive and negative delta SI groups based on our data (Table 2). Similar results were obtained after adjusting for confounding factors: high delta SI was an independent risk factor for both mortality and early mortality. Delta SI appeared to be an effective and efficient index of great relevance in rapid deterioration after ED admission. Conversely, high delta SI was not related to long HLOS in the regression analysis.

In this study, we further separated participants into subgroups for stratified analysis. High delta SI had higher odds ratios for mortality and early mortality in the elderly. Since progressive decline in various physiological functions has been noted in the elderly (28, 29), physiological stresses that were not serious at young ages can be life-threatening in old age (30). Fluctuations in HR and SBP are key factors for mortality among critically ill elderly individuals. Therefore, closely monitoring vital signs and of changes in delta SI is more important in elderly patients in situations of illness deterioration.

The subgroup analysis of vital signs highlights the importance of altered SBP and SI at triage; in patients with these parameters, high delta SI has higher odds ratio for early mortality. Patients with SBP > 100 mmHg at triage and with high delta SI present higher incidence of early mortality after ICU admission. This is consistent with previous studies that revealed that SI may be a predictor of mortality (31, 32) in critically ill patients. Since mortality in patients with shock with hypoperfusion remains high, as reported previously (19), we offer a more robust dynamic index to ensure that early intervention and management are available to these critically ill patients. While a high SI at ED triage was a predictor of mortality in previous studies (31, 33), aggressive resuscitation and close monitoring before ICU admission should be performed to avoid adverse outcomes.

Concerning the stratified analysis per diagnoses, we found that high delta SI in patients with sepsis is correlated with high mortality and early mortality. This corroborates the results of previous studies on the association between high SI and outcome in septic patients (12, 32). Similar results were not found for patients with diagnoses of respiratory failure or heart failure. Respiratory distress and respiratory failure could be unrelated to hypotension and HR alterations (34). In addition, patients with respiratory failure may need advanced airway ventilation or even mechanical ventilation, and the hemodynamic effects of mechanical breathing are quite complex (35). These factors might affect delta SI during ED management. Moreover, heart failure is caused by structural and functional defects in the myocardium, which result in impairment of ventricular filling or ejection of blood. Early stages of heart failure often lack specific signs, such as tachycardia or other classic presentations (36–38). Therefore, less association of delta SI with clinical outcomes can be presumed in heart failure patients who were admitted to the ICU.

Our study has several limitations. First, retrospective studies rely mostly on administrative data, which is limited by the information documented on medical records. Second, the study was conducted in two tertiary care EDs with similar systems; thus, the generalizability of these findings may be limited to comparable institutions. However, we believe that the number of patients analyzed in our article is enough to support our conclusions, and both studied hospitals nearly meet the highest medical standards in Taiwan. Further prospective studies should be conducted for a more precise analysis of our results; nevertheless, we believe that our research has laid good foundations for this research field. In conclusion, our results indicate that high delta SI may be greatly related to poor prognosis among critically ill patients, especially to early mortality. Elderly critically ill patients with poor vital signs at ED triage and a diagnosis of sepsis should be carefully monitored and assisted with prompt resuscitation and intensive treatment before admission to the ICU. We believe that it is crucial to monitor delta SI while managing patients and that delta SI could play an important role in clinical practice.

## Data Availability Statement

The raw data supporting the conclusions of this article will be made available by the authors, without undue reservation.

## Ethics Statement

The study protocol was approved by the institutional review board of both hospitals. All patients' and physicians' records and information were anonymized and de-identified before analysis.

## Author Contributions

Chi-FL: conceptualization and supervision. Y-SH, Chu-FL, I-MC, and Chi-FL: data curation and methodology. M-TT and Y-SH: formal analysis. M-TT, Y-SH, and Chi-FL: investigation. I-MC and Chi-FL: validation, writing—review, and editing. Y-SH and Chi-FL: visualization. Y-SH, I-MC, and Chi-FL: writing—original draft. All authors contributed to the article and approved the submitted version.

## Conflict of Interest

The authors declare that the research was conducted in the absence of any commercial or financial relationships that could be construed as a potential conflict of interest.
